# Mapping Cortical Degeneration in ALS with Magnetization Transfer Ratio and Voxel-Based Morphometry

**DOI:** 10.1371/journal.pone.0068279

**Published:** 2013-07-09

**Authors:** Mirco Cosottini, Paolo Cecchi, Selina Piazza, Ilaria Pesaresi, Serena Fabbri, Stefano Diciotti, Mario Mascalchi, Gabriele Siciliano, Ubaldo Bonuccelli

**Affiliations:** 1 Department of Neuroscience, University of Pisa, Pisa, Italy; 2 Unit of Neuroradiology, S.Chiara Hospital, Pisa, Italy; 3 Quantitative and Functional Neuroradiology Research Unit, Department of Biomedical Experimental and Clinical Sciences, University of Florence, Florence, Italy; University of Alberta, Canada

## Abstract

Pathological and imaging data indicate that amyotrophic lateral sclerosis (ALS) is a multisystem disease involving several cerebral cortical areas. Advanced quantitative magnetic resonance imaging (MRI) techniques enable to explore in vivo the volume and microstructure of the cerebral cortex in ALS. We studied with a combined voxel-based morphometry (VBM) and magnetization transfer (MT) imaging approach the capability of MRI to identify the cortical areas affected by neurodegeneration in ALS patients. Eighteen ALS patients and 18 age-matched healthy controls were examined on a 1.5T scanner using a high-resolution 3D T1 weighted spoiled gradient recalled sequence with and without MT saturation pulse. A voxel-based analysis (VBA) was adopted in order to automatically compute the regional atrophy and MT ratio (MTr) changes of the entire cerebral cortex. By using a multimodal image analysis MTr was adjusted for local gray matter (GM) atrophy to investigate if MTr changes can be independent of atrophy of the cerebral cortex. VBA revealed several clusters of combined GM atrophy and MTr decrease in motor-related areas and extra-motor frontotemporal cortex. The multimodal image analysis identified areas of isolated MTr decrease in premotor and extra-motor frontotemporal areas. VBM and MTr are capable to detect the distribution of neurodegenerative alterations in the cortical GM of ALS patients, supporting the hypothesis of a multi-systemic involvement in ALS. MT imaging changes exist beyond volume loss in frontotemporal cortices.

## Introduction

Amyotrophic lateral sclerosis (ALS) is a rapidly progressive neurodegenerative disorder characterized by upper motor neuron (UMN) and lower motor neuron (LMN) degeneration [1]. ALS diagnosis is based on clinical and electrophysiological findings, according to revised “El-Escorial” criteria [2].

The UMN involvement in ALS cannot be easily established based on the clinical ground because of the confounding effect of LMN involvement. The UMN damage has initially been explored mainly in the corticospinal tract using conventional [3], [4], [5], [6], [7] and advanced [8], [9], [10], [11], [12], [13] neuroimaging techniques. More recently, the research attention has been focused on the evaluation of the UMN at cortical level. Conventional MR studies subjectively evaluated the cortical morphology and signal changes in ALS patients [3], [5], [6], [7], [14], [15], but were limited by low specificity since similar findings are age related and can also be detected in subjects without ALS [16]. Voxel-based morphometry (VBM) is a quantitative automated method which performs a whole brain voxel-wise comparison of the local concentration of gray matter (GM) between two groups of subjects [17] and has been applied to the investigation of regional atrophy of the cerebral cortex in several neurodegenerative diseases. In ALS a reduction of GM volume within the precentral gyrus was reported by some authors [18], [19] and interpreted to reflect the classical pathological description of the loss of Betz cells in the V layer of the cerebral motor cortex [20]. Further VBM studies [18], [19], [21], [22] revealed that the cortical atrophy in ALS is not confined to the primary motor cortex but extends to premotor and parietal areas [18], [21] and to extramotor cortices such as temporal and prefrontal cortex [18], [19], [22]. The multisystem character of ALS is in line with the observation that about 2–3% of ALS patients develop frontotemporal dementia (FTD) [23], [24] and that in approximately 50% of ALS patients some cognitive impairment can be documented [25]. Notably atrophy in prefrontal and temporal cortex of ALS patients was described by pathological studies [26], [27], [28], [29], both in demented and non-demented ALS patients.

Magnetization Transfer Imaging (MTI) is a MR technique capable to explore the microstructure of the cerebral cortex in several neurodegenerative diseases [30], [31], [32], [33]. MTI creates a contrast between tissues by exploiting the phenomenon of magnetization exchange between the spins of free water and water bound to macromolecules. Magnetization Transfer ratio (MTr) is the simplest measure related to the efficiency of such exchange phenomena, which depends on the composition and integrity of the tissue examined [34]. Recently application of MTI to patients with ALS [35] revealed a cortical distribution of decreased MTr reflecting microstructural changes matching the distribution of the cortical damage known from the neuropathological examination including the precentral gyrus, superior frontal gyrus, middle frontal gyrus, frontal pole, superior parietal lobule, planum temporale and planum polare. Intriguingly, this distribution seems to correspond to the areas of atrophy reported in VBM studies [18], [19], [21].

In order to explore the topographical distribution of VBM and MTI changes in the cerebral cortex of ALS patients we performed a cross sectional study which included voxel based analysis (VBA) of T1 weighted images and MT ratio as well as a multimodal image analysis aimed to ascertain whether MT changes occur independently of regional volume loss.

## Materials and Methods

### Patients

We examined 18 consecutive right handed ALS patients (9 females and 9 males; mean age 55.6±10.7 years) and 18 right handed healthy controls (13 females and 5 males; mean age 48.8±10.8 years). Age and gender between groups were not significantly different (Mann-Withney U test p = 0.07 for age and Pearson Chi-square p = 0.17 for gender). All patients had definitive ALS diagnosis according to the revised El Escorial criteria [2], with clinical evidence of both UMN and LMN involvement. All patients were treated with riluzole (100 mg/die). Their clinical features are summarized in Table S1. Eleven patients showed the spinal form of the disease, 4 patients the bulbar form, 2 patients the flail arm form and 1 patient the flail leg form. The mean disease duration was 20.3±18.4 months. The mean score of ALS Functional Rating Scale revised (ALSFRS-r) [36] was 38.8±5.4 (maximum score: 48), the mean Medical Research Council scale (MRC) [37] at upper limb was 66.8±13.2 (maximum score: 80), at lower limb 58.1±10.9 (maximum score: 70). The UMN involvement was calculated on the basis of the presence (score 1) or absence (score 0) of hyperreflexia in the following regions: biceps (L+R), supinator (L+R), triceps (L+R), wrist (L+R), knee (L+R) and ankle (L+R). In addition, the presence (score 1) or the absence (score 0) of the Hoffmann sign (L+R) and the Babinsky sign (L+R) were also considered. The highest scores for each patients (maximum score: 16) reflected greater dysfunction of UMN. None of the ALS patients suffered from other neurological or systemic diseases. Control subjects were enrolled among spouses or friends of the patients. None had a history of psychiatric and neurological disorders and their clinical examination was unremarkable. All patients and controls gave informed consent to the MRI examination.

### Data Acquisition and Data Analysis

MR data were acquired on a 1.5 T scanner (Signa HDx, GE Healthcare, Milwaukee, Wisconsin) with high performance gradients (gradients strength 50 mT/m, maximum slew rate 150 T/m/s), equipped with an 8-channel head coil with ASSET-technology.

A high-resolution 3D spoiled gradient recalled (SPGR) sequence (repetition time (TR)/echo time (TE) = 28 ms/5 ms, flip Angle 40°, number of excitation 0.75, field of view (FOV) 240 mm, 192×192 matrix, 124 oblique-sagittal slices 1.5 mm thick, null gap) was acquired with (Sat) and without (No-Sat) the magnetization transfer (MT) saturation pulse. The MT pulse was a 1200 Hz off-resonance pulse.

The VBM analysis was carried out by the FSL-VBM optimized protocol [17] available in the FMRIB software library package (FSL). Brain Extraction Tool (BET) [38] was applied to No-Sat images to remove non-brain structures, then brain-extracted images were automatically segmented into GM, white matter (WM) and cerebrospinal fluid (CSF) tissue type by FMRIB’s Automated Segmentation Tool (FAST v4.0) [39]. In order to create a study-specific gray matter template, all the GM volume images were affine registered to the Montreal Neurological Institute (MNI) 152 standard space [40] using FMRIB’s Linear Image Registration Tool (FLIRT) [41], [42] and averaged. Then, the native GM images were non-linearly re-registered to the study specific template using FMRIB’s Nonlinear Image Registration Tool (FNIRT) [43], [44]. The registered GM images were modulated by dividing by the Jacobian of the warp field (to correct for local expansion or contraction due to the non-linear registration) and then smoothed with an isotropic Gaussian kernel with a sigma of 3 mm. A voxel-wise general linear model (GLM) was applied using permutation-based non-parametric testing (5000 permutations) and Threshold-Free-Cluster-Enhancement (TFCE) [45], avoiding the use of an arbitrary threshold for the initial cluster-formation. The significance level was set at p<0.05 corrected for multiple comparisons via Family-wise Error (FWE) correction across space. Since voxel based quantification of atrophy is influenced by aging [46] we inserted age and gender of patients and controls as covariate variables within the GLM matrix.

MT image processing was performed as described in a previous work [35]. Briefly, a fully automated procedure implemented by FSL software package (4.4 version) [17] was used. The analysis included brain extraction in native Sat images by using BET and affine registration by using FLIRT of all the Sat images to corresponding No-Sat images to obtain the best alignment between the two images. MTr images were calculated voxel-wise by the following formula: MTr = 100 X (NoSat - Sat)/NoSat. Then, we applied a thresholding on MTr images assuming that values of MTr greater than 75% were due to image noise or partial volume effects [47]. Non-brain tissues masks obtained from BET segmentation of No-Sat images were applied onto MTr images to obtain MTr images of cerebral cortex for each subject. Similarly to what previously done for the VBM analysis, MTr images masked with GM maps (GM MTr) were aligned to MNI standard space and averaged to create a reference template for MTr images. Then, all GM MTr images were non-linearly re-registered to the study specific template, divided by the Jacobian of the warp field and smoothed with an isotropic Gaussian kernel with a sigma of 3 mm. The statistical analysis was performed with a voxel-based analysis (VBA) (instead of a ROI approach as done in a previous work [35]). A voxel-wise GLM was applied using permutation-based non-parametric testing (5000 permutations) and TFCE with a significance level set at p<0.05 FWE corrected for multiple comparisons. Age and gender of patients and controls were inserted as covariate variables in the model.

In order to exclude the possibility that differences found in voxel-wise MTr analysis were due to brain atrophy in ALS patients compared to healthy subjects, we performed a multimodal image analysis [48]. To this aim we used GM volumetric maps as voxel-wise nuisance regressors and incorporated them in the GLM analysis of the MTr maps obtaining for each voxel a different design matrix [49]. A permutation-based non parametric test (5000 permutations) and TFCE with a significance level set at p<0.05 FWE corrected for multiple comparisons was thus applied.

In order to establish a correlation among clinical variables and VBM or MTr changes, ALSFRS score, MRC for upper limb, MRC for lower limb, disease duration and upper motor neuron sign score was inserted as covariate variables within different GLM design matrix.

Statistical maps were superimposed onto Anatomical Automated Labeling (AAL) Atlas [50] in order to localize areas of atrophy and MTr changes.

### Ethics Statement

The research was conducted according to the principles expressed in the Declaration of Helsinki. The study was approved by the Ethics Commitee of Azienda Ospedaliera Universitaria Pisana, Pisa, Italy. Written informed consent was obtained from all the study partecipants.

## Results

### VBM

**Figure 1:**
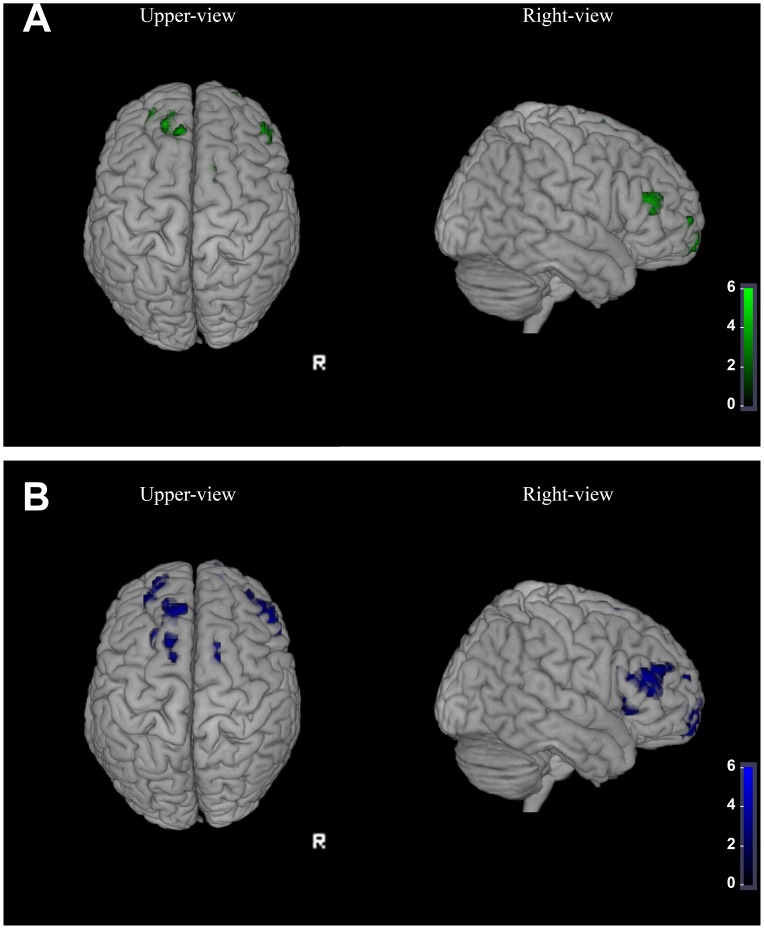
Regional atrophy and MTr decrease in ALS. (A) Results of the between group VBM analysis [p<0.05 FWE corrected for multiple comparisons] superimposed on volume rendering template in the right and upper views reveals clusters (in green) of gray matter atrophy. Clusters are located in motor related areas but also in extra-motor prefrontal and temporal cortex in line with the hypothesis of a multi-systemic involvement in ALS. (B) Results of the between group MTI analysis [p<0.05 FWE corrected for multiple comparisons] superimposed on volume rendering template reveals clusters (in blue) of reduced MTr in motor related and extra-motor cortical areas indicating microstructural changes in the cerebral cortex of ALS patients.

Results of the between group VBM analysis revealed several clusters of reduced cortical GM in ALS patients compared to healthy controls (p<0.05 FWE corrected for multiple comparisons). They were located in the superior, middle and inferior frontal gyri (AAL 3,4,6,7,8,14,23,26), in the supplementary motor area (AAL 20) and in the temporal lobe (AAL 85) (Figure 1A and Table S2). These regional GM volume losses were bilateral but more extended on the right hemisphere.

### MTI

Results of the between group MTI analysis revealed that the MTr values were significantly lower in ALS patients than in the healthy controls in the following cortical regions: superior, middle and inferior frontal gyrus (AAL 3,4,6,7,8,9,10,11,12,13,14,15,16,23,24,26), gyrus cinguli (AAL 31,32), supplementary motor area (AAL 19,20), insula (AAL 29,30) and temporal lobe (AAL 38,40,42,56,86) (Figure 1B and Table S3).

### Multimodal Imaging Analysis

**Figure 2:**
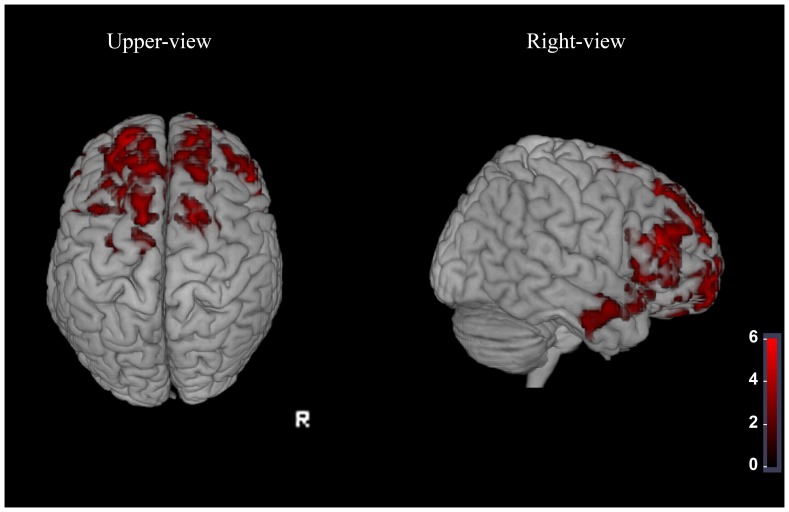
Regional MTr decrease surviving correction for local atrophy in ALS. Results of the between group MTI analysis adjusted for atrophy [p<0.05 FWE corrected for multiple comparisons] superimposed on volume rendering template in the right and upper views reveals clusters (in red) of reduced MTr mainly affecting premotor and frontotemporal cortex. MTI changes exist beyond volume loss in these cortical.

Results of the between group regional MTI analysis corrected for atrophy revealed that the MTr values were significantly lower in ALS patients than in the healthy controls in the superior, middle and inferior frontal gyri (AAL 3,4,5,6,7,8,9,10,11,12,13,14,15,16,17,23,24,25,26,27,28), in the gyrus cinguli (AAL 31,32,33,34), in the supplementary motor area (AAL 19,20), in the insula (AAL 29,30) and in the temporal lobe (AAL 37,38,40,42,56,82,83,84,86,87,88,90) (Figure 2 and Table S4).

No cluster of significant association between atrophy or MTr changes and clinical scales was detected in the respective statistical maps.

## Discussion

ALS is emerging as a multi-system disease involving several frontal-temporal structures beside motor structures and functions [51]. Our results revealing clusters of atrophy and MTr reduction not only in not primary motor cortices but also in extramotor areas are in line with such a multi-systemic nature of ALS [35]. Moreover the atrophy and MTr decrease in prefrontal and temporal cortex along with other neuroimaging [18], [19], [21], [22], [52], [53], [54], [55], functional [56], [57] and pathological data [26], [27], [28], [58], [59] support the clinical [60], [61] and immuno-histochemical [62] overlap between ALS and FTD which can be considered a clinico-pathological spectrum described with the label of TDP-43 proteinopathies [63].

In order to compare the spatial distribution of atrophy and MTr changes in ALS we adopted in the present study a voxel based approach of MT data [64], [65] instead of a ROI based analysis. Due to the large number of multiple comparison, VBA increases the likelihood of type I error with a reduction of statistical power with respect to the ROI analysis. The resulting lower sensitivity of VBA may be capable of revealing only regions where more pronounced MTr changes are present. This notwithstanding, we detected cluster of MTr decrease in motor-related and extra-motor cortical areas, but not in the primary motor cortex, confirming and increasing the robustness of the results obtained with a ROI approach [35]. Moreover VBA enable a close spatial comparison of VBM and MTI with additional correction of MTr results for the local cerebral atrophy [48].

Since a region specific MTr decrease in the sensory motor cortex is observed with advancing age [46], we inserted the age as a covariate variable in the GLM in order to limit the influence of this phenomena onto the cortex that is primarily affected in ALS.

Although we do not know the exact meaning of the reduced MTr in the cerebral motor-related and extra-motor cortex of ALS patients, the rate of MT effect depends on the surface chemistry and the biophysical dynamics of the macromolecules, as well on the concentration of macromolecules and water [66] and it seems to be strongly associated with the degree of tissue damage [67]. It is hence reasonable to suppose that tissutal microstructural changes related to neurodegenerative phenomena [68], [69] are responsible of decreased MTr in the cerebral cortex of ALS patients.

The neuronal loss and degeneration in the V layer of the cortex into the precentral gyrus is considered the pathological hallmark of the UMN involvement in ALS [20]. The local decrease of the GM volume revealed by several VBM studies [18], [19], [22] and by manual or automated measurements of the thickness of the cortical layer [48], [51] along with MTr decrease might be regarded as structural surrogates of UMN degeneration. Moreover it has been reported that the precentral gyrus is the site of combined atrophy and reduced blood oxygen level-dependent (BOLD) activity during a controlateral handgrip movement [19]. The BOLD signal reduction decreased parallel to the loss of motor function [19], [70] and both worsened over time (personal unpublished data) suggesting a progressive functional and structural impairment in the primary motor cortex of ALS patients.

In the present study the voxel-wise analysis of GM volume, MTr and MTr data corrected for regional atrophy failed to show any structural abnormality in the precentral gyrus of ALS patients. This result may reflect both the fact that the pathological changes in primary motor cortex of ALS patients are subtle [71] and the adopted statistical analysis and thresholds in VBA are too conservative for revealing mild microstructural changes. Indeed most of VBA studies revealing atrophy in primary motor cortex of ALS patients utilized less conservative statistics. In particular data were uncorrected for multiple comparisons [22], [70] or evaluated within sub-volume of the whole brain (namely small volume correction) [18], [19], [54]. Notably, in line with the result of the present study, the only study which compared ALS patients and controls by using a VBM corrected for multiple comparison in a whole brain fashion [55] failed to reveal primary motor cortex atrophy confirming the view that the latter is a subtle phenomena detectable only with less conservative statistics.

On the other hand our and other studies showed clusters of atrophy and MTr changes in motor-related area. Clusters of decreased MTr independent of cortical atrophy were observed in the premotor cortex particularly in the superior, middle frontal gyri pertaining to the fronto parietal circuit [72] that is implicated in the transformation of sensory information into actions [73]. This GM atrophy and MTr reduction in the prefrontal-related dorsal premotor areas that are typically hypo-activated in ALS patients during execution of motor tasks [19], could represent the structural correlate of neuronal degeneration in motor-related areas, in agreement with previous hystopathological studies [74].

At multimodal image analysis, the MTr decrease in prefrontal and temporal cortex of our ALS patients survived correction for local atrophy indicating that the microstructural changes exist beyond the areas of cortical atrophy. This interpretation is supported by the observation that MTI can detect microstructural changes in cortical areas unaffected by atrophy also in patients with Alzheimer disease (AD) [30], [32] and Huntington disease [33]. Recently, a quantitative magnetization transfer index, measured with a VBA corrected for atrophy, was reported to be reduced in hippocampus, temporal lobe, posterior cingulate and in the parietal cortex of patients with AD [48]. The Authors submitted that MT parameters might provide complementary information to VBM in the characterization of the neurodegenerative processes occurring in AD, and this is likely the case also in other neurodegenerative diseases.

A longitudinal evaluation of VBM and MTr data could be a further fundamental step in evaluating their spatial and temporal relationship in the progression of motor and cognitive functions in ALS and is currently under way.

We recognize as main limitation of our work that we did not obtain a detailed neuropsychological profile of our patients. This hinders investigation of the relationship between behavioural and cognitive functions and regional volume or MTr of the cortical GM in specific brain structures [68], [69].

### Conclusions

VBM and MTI reveal regional atrophy and microstructural changes of the cerebral cortex in ALS involving not only motor-related areas but also frontotemporal cortices.

MTr reduction occurs in non atrophic cortical areas suggesting that microstructural degenerative phenomena can extend beyond the tissue loss in ALS.

## Supporting Information

Table S1 Demographic and Clinical data.
(DOC)

Table S2 VBM results: clusters of cortical atrophy.
(DOC)

Table S3 MT Imaging results: clusters of reduced MT ratio.
(DOC)

Table S4 MT Imaging results: clusters of reduced MT ratio adjusted for atrophy.
(DOC)
