# Supplementary material for: Mapping Cortical Degeneration in ALS with Magnetization Transfer Ratio and Voxel-Based Morphometry
Source: PLoS One. 2013 Jul 9;8(7):e68279. doi: 10.1371/journal.pone.0068279 (PMC3706610; doi:10.1371/journal.pone.0068279)
Supplement: Table S1 — Demographic and Clinical data. (DOC) [file pone.0068279.s001.doc]

**Table S1. Demographic and Clinical data.**

| **Patient #** | **Age** | **Sex** | **ALS form** | **Disease duration (months)** | **ALSFRS-r score** | **MRC scale upper limb** | | | **MRC scale lower limb** | | | **UMN** |
| --- | --- | --- | --- | --- | --- | --- | --- | --- | --- | --- | --- | --- |
|  |  |  |  |  |  | **total** | **R** | **L** | **total** | **R** | **L** |  |
| 1 | 61 | F | B | 16 | 41 | 76 | 40 | 36 | 67 | 35 | 32 | 14 |
| 2 | 57 | F | S | 12 | 33 | 55 | 29 | 26 | 46 | 20 | 26 | 0 |
| 3 | 53 | F | S | 4 | 42 | 76 | 36 | 40 | 64 | 29 | 35 | 14 |
| 4 | 71 | M | S * | 30 | 35 | 46 | 24 | 22 | 63 | 31 | 32 | 0 |
| 5 | 59 | M | S ° | 38 | 35 | 69 | 33 | 36 | 42 | 19 | 23 | 9 |
| 6 | 66 | M | B | 14 | 37 | 64 | 32 | 32 | 70 | 35 | 35 | 0 |
| 7 | 55 | M | S | 5 | 46 | 80 | 40 | 40 | 57 | 27 | 30 | 2 |
| 8 | 49 | F | S | 12 | 39 | 72 | 40 | 32 | 60 | 33 | 27 | 16 |
| 9 | 66 | M | S | 16 | 42 | 66 | 34 | 32 | 55 | 29 | 26 | 8 |
| 10 | 38 | M | S * | 26 | 34 | 45 | 23 | 22 | 59 | 30 | 29 | 7 |
| 11 | 63 | M | S | 67 | 28 | 42 | 21 | 21 | 28 | 14 | 14 | 0 |
| 12 | 49 | M | S | 62 | 30 | 52 | 26 | 26 | 56 | 28 | 28 | 14 |
| 13 | 52 | M | S | 4 | 41 | 75 | 35 | 40 | 62 | 28 | 34 | 1 |
| 14 | 63 | F | B | 15 | 41 | 80 | 40 | 40 | 65 | 30 | 35 | 16 |
| 15 | 33 | F | S | 6 | 47 | 77 | 37 | 40 | 66 | 33 | 33 | 16 |
| 16 | 40 | F | S | 13 | 45 | 68 | 28 | 40 | 66 | 31 | 35 | 8 |
| 17 | 69 | F | B | 10 | 40 | 80 | 40 | 40 | 70 | 35 | 35 | 2 |
| 18 | 56 | F | S | 15 | 43 | 80 | 40 | 40 | 50 | 29 | 21 | 0 |
| Mean | 55.6 |  |  | 20.3 | 38.8 | 66.8 | 33.2 | 33.6 | 58.1 | 28.7 | 29.4 | 7.1 |
| SD | 10.7 |  |  | 18.4 | 5.4 | 13.2 | 6.6 | 7.2 | 10.9 | 5.7 | 5.9 | 6.5 |

* Flail Arm form

° Flail Leg form

Abbreviations: ALS = Amyotrophic Lateral Sclerosis; ALSFRS-r = ALS Functional Rating Scale-revised; B = Bulbar; L = Left; MRC scale = Medical Research Council scale; R = Right; S = Spinal; SD = Standard Deviation; UMN = Upper Motor Neuron score.
