# Supplementary material for: Mapping Cortical Degeneration in ALS with Magnetization Transfer Ratio and Voxel-Based Morphometry
Source: PLoS One. 2013 Jul 9;8(7):e68279. doi: 10.1371/journal.pone.0068279 (PMC3706610; doi:10.1371/journal.pone.0068279)
Supplement: Table S2 — VBM results: clusters of cortical atrophy. (DOC) [file pone.0068279.s002.doc]

**Table S2.** VBM results: clusters of cortical atrophy.

| **Anatomical location** | **Side** | **AAL number** | **Cluster size** | **T max** | **MNI Coordinates (mm)** | | |
| --- | --- | --- | --- | --- | --- | --- | --- |
|  |  |  | **(mm^3^)** |  | **x** | **y** | **z** |
| **FRONTAL LOBES** |  |  |  |  |  |  |  |
| Frontal Sup | R L | 3,4,6,20 | 1600 | 5.6 | -18 | 44 | 48 |
| Frontal Mid | R L | 7,8,23,26 | 2216 | 5.1 | 12 | 64 | -6 |
| Frontal Inf | R | 14 | 728 | 5.5 | 46 | 34 | 14 |
| **TEMPORAL LOBES** |  |  |  |  |  |  |  |
| Temporal Mid | L | 85 | 320 | 6.2 | -52 | -36 | 2 |

Abbreviations: AAL = Automated Anatomical Labeling atlas; L = Left; MNI = Montreal Neurological Institute standard space; R = Right.
