# Supplementary material for: Mapping Cortical Degeneration in ALS with Magnetization Transfer Ratio and Voxel-Based Morphometry
Source: PLoS One. 2013 Jul 9;8(7):e68279. doi: 10.1371/journal.pone.0068279 (PMC3706610; doi:10.1371/journal.pone.0068279)
Supplement: Table S3 — MT Imaging results: clusters of reduced MT ratio. (DOC) [file pone.0068279.s003.doc]

**Table S3. MT Imaging results: clusters of reduced MT ratio.**

| **Anatomical location** | **Side** | **AAL number** | **Cluster size** | **T max** | **MNI Coordinates (mm)** | | |
| --- | --- | --- | --- | --- | --- | --- | --- |
|  |  |  | **(mm^3^)** |  | **x** | **y** | **z** |
| **FRONTAL LOBES** |  |  |  |  |  |  |  |
| Frontal Sup | R L | 3,4,6,19,20,23,24 | 5872 | 5.0 | 10 | 12 | 60 |
| Frontal Mid | R L | 7,8,9,10,26 | 5416 | 4.2 | 48 | 34 | 18 |
| Frontal Inf | R L | 11,12,13,14,15,16 | 6192 | 4.7 | 48 | 32 | 16 |
| Insula | R L | 29,30 | 1584 | 4.0 | 34 | 18 | 0 |
| Cingulum | R L | 31,32 | 3376 | 5.2 | 4 | 32 | 12 |
| **TEMPORAL LOBES** |  |  |  |  |  |  |  |
| Hippocampus | R | 38,40 | 4816 | 4.7 | 24 | -22 | -14 |
| Amygdala | R | 42 | 680 | 3.7 | 24 | -8 | -12 |
| Temporal Mid | R | 86 | 352 | 4.3 | 50 | 0 | -20 |
| Temporal Inf | R | 56 | 112 | 3.1 | 26 | -30 | -24 |

Abbreviations: AAL = Automated Anatomical Labeling atlas; L = Left; MNI = Montreal Neurological Institute standard space; R = Right.
