# Supplementary material for: Mapping Cortical Degeneration in ALS with Magnetization Transfer Ratio and Voxel-Based Morphometry
Source: PLoS One. 2013 Jul 9;8(7):e68279. doi: 10.1371/journal.pone.0068279 (PMC3706610; doi:10.1371/journal.pone.0068279)
Supplement: Table S4 — MT Imaging results: clusters of reduced MT ratio adjusted for atrophy. (DOC) [file pone.0068279.s004.doc]

**Table S4.** MT Imaging results: clusters of reduced MT ratio adjusted for atrophy.

| **Anatomical location** | **Side** | **AAL number** | **Cluster size** | **T max** | **MNI Coordinates (mm)** | | |
| --- | --- | --- | --- | --- | --- | --- | --- |
|  |  |  | **(mm^3^)** |  | **x** | **y** | **z** |
| **FRONTAL LOBES** |  |  |  |  |  |  |  |
| Frontal Sup | R L | 3,4,5,6,19,20,23,24 | 39264 | 6.1 | 16 | 58 | 18 |
| Frontal Mid | R L | 7,8,9,10,25,26 | 30344 | 7.1 | -38 | 18 | 36 |
| Frontal Inf | R L | 11,12,13,14,15,16,17,27,28 | 31944 | 6.2 | -38 | 18 | 34 |
| Insula | R L | 29,30 | 5064 | 4.7 | 36 | 18 | 14 |
| Cingulum | R L | 31,32,33,34 | 9568 | 6.8 | 14 | 44 | 14 |
| **TEMPORAL LOBES** |  |  |  |  |  |  |  |
| Hippocampus | R L | 37,38,40 | 8328 | 5.5 | 38 | -24 | -10 |
| Amygdala | R | 42 | 1208 | 4.0 | 24 | 2 | -12 |
| Temporal Sup | R L | 82,83,84 | 1416 | 3.7 | -52 | 12 | -24 |
| Temporal Mid | R L | 86,87,88 | 3664 | 4.7 | 50 | 0 | -24 |
| Temporal Inf | R | 56,90 | 1776 | 3.7 | 54 | -14 | -24 |

Abbreviations: AAL = Automated Anatomical Labeling atlas; L = Left; MNI = Montreal Neurological Institute standard space; R = Right.
